# Impact of Fermented Foods on Human Cognitive Function—A Review of Outcome of Clinical Trials

**DOI:** 10.3390/scipharm86020022

**Published:** 2018-05-31

**Authors:** Bhagavathi Sundaram Sivamaruthi, Periyanaina Kesika, Chaiyavat Chaiyasut

**Affiliations:** Innovation Center for Holistic Health, Nutraceuticals, and Cosmeceuticals, Faculty of Pharmacy, Chiang Mai University, Chiang Mai 50200, Thailand; p.kesika@gmail.com

**Keywords:** fermented food, functional food, cognition, memory, anxiety

## Abstract

Food is an essential need for all living creatures which provides the energy to maintain life and grow further. Fermentation is a process used to preserve and advance the quality of foods, and those foods are known as fermented foods. Some foods offer health benefits to consumers apart from nutrition, and such foods are called as functional foods. Most functional foods are fermented foods, and the fermenting microorganism plays a precious role in the functional property of the food. Cognitive decline is closely associated with the productivity of an individual and the society. Even though cognitive decline is connected to aging, dietary pattern influences memory, anxiety and other social behaviors. Many scientific studies have explained the link between food habits and cognitive functions by in vitro and in vivo models. The present review compiled the clinical data on the impact of fermented foods on human cognitive function.

## 1. Introduction

The cognition of a person is defined as a collection of mental processes such as perception, instinct, and reasoning, by which knowledge is attained to survive in the society. Age is the primary factor that affects cognitive function. The decline in cognition is linked with increased dependence on others, which affects quality of life. Even though the memory decline begins at 20 to 30 years of age, the visible or noticeable memory loss which happens after 60 years of age affects working memory (such as a decline in processing speed, and spatial management) [1,2]. Age-related cognitive failure is often associated with the development of mild to severe cognitive damage or dementia, which is one of the burdening health issues among the world elderly population. The prevention of cognitive decline or betterment of cognitive function among older people is necessary to enhance the well-being of their life. 

Fermented foods (FFs) were used by our ancestors without knowing the contribution of spontaneous fermentation, which improved the functional quality of food. FFs are diverse, based on the origin of the food, raw materials, processing, and even depending on the person who prepares the food. Some of the FF preparations were preserved as family secret, whereas salting and drying are the conventional methods of food preservation in the ancient period. Fermentation was employed unknowingly, without any scientific knowledge of the fermentation process, to preserve the food for future use, especially during winter or drought periods [3].

The fermented plant-based foods, such as fermented vegetables, and fruits, play a significant role in human nourishment, providing vitamins, minerals, trace elements, and other essential nutrients. Fermentation of plant-based foods is a general way to preserve and expand the nutritional and sensory features of food. Inziangsang, Pak-Gard-Dong, Dakguadong and Burong mustala (fermented mustard leaf), Jiang-gua and Khalpi (fermented cucumber), Gundruk (fermented cauliflower, radish, cabbage, mustard), Soidon (fermented bamboo shoot), Ca muoi (fermented eggplant), Nozawana-Zuke (fermented turnip), Paocai, Sauerkraut, Sayur asin and Suan-tsai (fermented cabbage), Yan-tsai-shin (fermented broccoli), Yan-jiang (fermented ginger), Yan-taozih (fermented peaches), Kimchi (fermented cabbage), and gochujang (fermented spicy red pepper paste) were generally-used fermented plant-based foods of Asian countries, especially in respective countries such as India, Thailand, China, Taiwan, Philippines, Vietnam, Japan, and Korea [3,4,5,6,7,8,9,10,11,12,13,14,15,16,17].

Foods with additional health benefits such as the ability to reduce the disease risk or endorse the optimal health are defined as functional foods, as per the American Dietetic Association [18]. The functional food market value was $129.39 billion in 2015, and it has been projected to increase up to ~$230 billion in 2024. The market for functional food intended to improve mental health and to increase the memory power is rapidly growing. Several scientific reports suggested that the fermented plant foods improved the cognitive function with in vitro and in vivo models [19,20,21,22,23,24]. Several reports suggested that fermented plant juices can be used in cosmetic preparations [25] and can be potent nutraceutical products [26]. Several bioactive probiotic strains were isolated from dairy products [27,28], and the microbial diversity and its functional properties were reported as one of the key factors of the health-promoting property of the food. However, human clinical trials on the impact of supplementation of FF on cognitive function is limited, and the results are not consistent. 

The present review summarizes the effect of FFs on cognitive function based on recent scientific evidence.

## 2. Fermented Foods and Cognition

The summary and results derived from the clinical studies, in the correlation between FF consumption and cognitive function, have been listed in Table 1.

Since only a few clinical studies have been reported regarding the impact of fermented foods on cognitive function, a few epidemiological studies were also discussed in the present review. Several studies have reported that fermented soybean product (tempeh) does not show a negative impact on cognitive function, revealing the beneficial effect of fermentation [29,30]. Studies among older adults (719 people of 52–98 years old) of Indonesian villages (Borobudur and Sumedang) and urban areas (Jakarta) suggested that the higher consumption of tofu, tempeh (fermented soybean product indigenous to Indonesia), and tahoe worsen the memory. Particularly, a high consumption of tofu, soybean curd, reduces the memory. The researcher suggested that the presence of phytoestrogen may be associated with memory loss among the tofu-consuming elderly people of Indonesia, while tempe contains folate, and its consumption did not affect the memory of the studied people [29]. A follow-up study was conducted with the elderly people of Borobudur [30], who have initially participated in a 2008 study conducted by Hogervorst et al. [29] The information on the cognitive function of about 142 people were analyzed. The revisit study suggested that the people who had memory decline during the initial study were no longer associated with tofu-mediated dementia. The lifestyle and dietary changes improved the health status of the young and middle-aged people, but not the elderly people. The study also proposed that estrogenic compounds did not affect the cognition and brain function of the younger people [30].

The impact of supplementation of *Lactobacillus helveticus* IDCC3801 mediated fermented milk (LHFM) on the cognitive function of Korean healthy elderly people was investigated. The experiment group was supplemented with different concentrations (0.5, 1, and 2 g) of LHFM in tablet form while the control group consumed the same amount of a tablet which contained no LHFM for 12 weeks.

The verbal-learning test, digit-span test, and story recall tests were performed to assess the cognitive function. The perception of stress was determined by perceived stress scale (PSS) and geriatric depression scale-short form (GDS-SF: a questionnaire-based tool to measure depression in older adults) tests. The brain-derived neurotrophic factor (BDNF) and whole blood viscosity (WBV) were used as biomarkers to measure the changes in cognition during the study. The results revealed that consumption of LHFM for three months improved the cognitive function of healthy older adults, which was proved by the cognitive tests. Significant changes were found to be observed in biomarkers (BDNF, and WBV), and Self-rating scales (PSS, and GDS-SF) [31].

The impact of supplementation (190 g per day for 8 weeks) of *L. helveticus* CM4 mediated fermented milk on cognitive function of Japanese middle-aged healthy adults was investigated. The neuropsychological assessment was performed using the repeatable battery for the assessment of neuropsychological status (RBANS) test to evaluate the cognitive function of the participants (Table 1). The study results indicated that the consumption of *L. helveticus* CM4 mediated fermented milk effectively improved the cognitive function of the Japanese middle-aged healthy adults [32].

The effect of probiotic milk (*Bifidobacterium bifidum*, *L. casei*, *L. fermentum*, and *L. acidophilus*; dose: 2 × 10^9^ CFU per gram of each probiotic per day) supplementation (for 12 weeks) on the cognitive function of the Alzheimer’s disease patients with cognitive impairment was investigated. Assessment of cognitive function was performed in the participants using the mini-mental state examination (MMSE) test. The intervention of probiotic milk for 12 weeks showed a positive effect on the cognitive function of the Alzheimer’s disease patients [33].

A cross-sectional study was conducted by Hilimire et al. [34] to determine the association of FF consumption, particularly probiotic based foods, and social anxiety symptoms. About 710 ethnically varied people (18–38 years old, including 445 females) were involved in the study. The social phobia and anxiety record (a test to measure social anxiety), the big five personality inventories (a behavior inventory with five different domains such as extraversion, amicability, meticulousness, honesty, and neuroticism), food and exercise frequencies were recorded and analyzed. The study results suggested that the higher frequency of consumption of FFs reduces the symptoms of social anxiety. Particularly, probiotic-based FF consumption improved cognitive function and decreased social anxiety among people who have a genetic risk of neuroticism [34].

The influence of dietary patterns (DPs) on cognitive decline in Chinese elderly people were determined in a study. A questionnaire has been prepared to assess the dietary pattern of the subjects. According to the Taiwan Nutritional Content database, about 44 food items were categorized into 24 food groups, and people were asked to respond to the questions with a scaling system. The cognition, both global and domestic, was evaluated by the Montreal cognitive assessment—Taiwan version. The serum level of folate and vitamin B12 were also determined. Traditional meat and vegetable-based food habits were found as three major DPs among the study group. The analysis revealed that vegetable and traditional food consumption reduces the risk of logical memory decline, while high meat intake reduces verbal fluency. The study revealed that the DPs were allied with different cognitive domains of Chinese elderly people [35].

## 3. Conclusion and Future Prospectus

As per our literature survey, an insufficient number of clinical trials were found about the impact of FF on cognitive function. The results were not consistent among ethnic groups. Most of the studies—five out of six—were conducted among Asian people. The high consumption of soy product, tofu, was associated with cognitive decline and was remedied in the young and middle-aged population by changing the food habits. The intake of probiotic-containing FF helps to advance cognitive function. Altogether, the results of the clinical studies suggested that dietary pattern is closely associated with cognitive function, which is influenced by age, ethnicity, amount of food, nature of fermentation, etc. The traditional method of FF preparation is also a critical factor that was proved among the Chinese population. Further, detailed clinical investigations are required with proper follow-up studies to explore the impact of consumption of FFs on the cognitive function of an individual.

## Figures and Tables

**Table 1:** Clinical outcome of fermented food (FF) supplementation on cognitive function.

S. No	Fermented Food	Experimental Subjects	The Geographical Region of the Study	Assessments	Result	Ref.
1	Tofu, tempeh, or tahoe and other genistein-containing foods	115 elderly people (Age: 52–98 years old)	Indonesia (Borobudur)	Psychiatric assessment, depression scale based on the Cambridge Mental Disorder of the Elderly Examination	The consumption of soy-based FF improved the memory and cognitive function of middle-aged people but not the elderly people. There was no negative impact on the brain cells and cognitive function.	[30]
2	*Lactobacillus helveticus* IDCC3801 mediated fermented milk	Elderly people (Age: 60–75 years old)	Korea	Self-rating scales, Cognitive tests, and Biomarker analysis	The consumption of fermented milk improved the cognitive function of the elderly people.	[31]
3	*L. helveticus* CM4 mediated fermented milk	Healthy adults (middle age)	Japan	Neuropsychological assessment (repeatable battery for the assessment of neuropsychological status (RBANS) test)	The consumption of fermented milk improved the cognitive function of middle-aged healthy adults.	[32]
4	Probiotic milk (*Bifidobacterium bifidum*, *L. casei*, *L. fermentum*, *L. acidophilus*)	60 Alzheimer’s disease patients (Age: 60–95 years old)	Iran	Cognitive assessment (mini-mental state examination (MMSE) test)	The consumption of probiotic milk improved the cognitive function of the Alzheimer’s disease patients.	[33]
